# Exploring Long-Read Metagenomics for Full Characterization of Shiga Toxin-Producing *Escherichia coli* in Presence of Commensal *E. coli*

**DOI:** 10.3390/microorganisms11082043

**Published:** 2023-08-09

**Authors:** Sandra Jaudou, Carlus Deneke, Mai-Lan Tran, Carina Salzinger, Fabien Vorimore, André Goehler, Elisabeth Schuh, Burkhard Malorny, Patrick Fach, Josephine Grützke, Sabine Delannoy

**Affiliations:** 1 COLiPATH Unit, Laboratory for Food Safety, ANSES, 94700 Maisons-Alfort, France; sandra.jaudou.ext@anses.fr (S.J.);; 2 National Study Center for Sequencing in Risk Assessment, Department of Biological Safety, German Federal Institute for Risk Assessment, 12277 Berlin, Germany; 3 Genomics Platform IdentyPath, Laboratory for Food Safety, ANSES, 94700 Maisons-Alfort, France; 4 National Reference Laboratory for *Escherichia coli* Including VTEC, Department of Biological Safety, German Federal Institute for Risk Assessment, 12277 Berlin, Germany

**Keywords:** metagenomics, STEC, milk, long-read sequencing, isolation-free characterization

## Abstract

The characterization of Shiga toxin-producing *Escherichia coli* (STEC) is necessary to assess their pathogenic potential, but isolation of the strain from complex matrices such as milk remains challenging. In previous work, we have shown the potential of long-read metagenomics to characterize *eae*-positive STEC from artificially contaminated raw milk without isolating the strain. The presence of multiple *E. coli* strains in the sample was shown to potentially hinder the correct characterization of the STEC strain. Here, we aimed at determining the STEC:commensal ratio that would prevent the characterization of the STEC. We artificially contaminated pasteurized milk with different ratios of an *eae*-positive STEC and a commensal *E. coli* and applied the method previously developed. Results showed that the STEC strain growth was better than the commensal *E. coli* after enrichment in acriflavine-supplemented BPW. The STEC was successfully characterized in all samples with at least 10 times more STEC post-enrichment compared to the commensal *E. coli*. However, the presence of equivalent proportions of STEC and commensal *E. coli* prevented the full characterization of the STEC strain. This study confirms the potential of long-read metagenomics for STEC characterization in an isolation-free manner while refining its limit regarding the presence of background *E. coli* strains.

## 1. Introduction

Shiga toxin-producing *Escherichia coli* (STEC) are diarrheic *E. coli* characterized by the presence of a specific virulence factor: the Shiga toxin [1]. A subset of STEC, known as *eae*-positive STEC, additionally possesses the ability to adhere to the intestinal epithelium and cause attaching and effacing (A/E) lesions. Adhesion is conferred by the intimin protein encoded by the *eae* gene located on the locus of enterocyte effacement (LEE) pathogenicity island [2]. EFSA reports showed that *eae*-positive STEC are most frequently associated with severe human illnesses, especially hemolytic uremic syndrome (HUS) [3].

STEC detection methods in food products involve time-consuming isolation steps to characterize the STEC strain [4]. Isolation-free approaches for STEC characterization in food matrices would circumvent this but imply using metagenomics [5,6]. While short-read metagenomics enables STEC detection from complex matrices, it does not permit full characterization of the strain. In addition, different *E. coli* strains may be present in the same sample [7,8]. Thus, strain-level metagenomics for the identification of STEC such as *eae*-positive STEC or carrying other adhesion factors is challenging. Long-read metagenomics have been shown to be applicable for identifying *eae*-positive STEC in artificially contaminated beef, raw milk, and wastewater using different bio-informatics approaches [9,10,11].

In a previous study, we tested the feasibility of identifying an *eae*-positive STEC strain from artificially contaminated raw milk samples using long-read metagenomics. Assembly-based methods have the advantage of enabling the characterization of the STEC strain but depend on the contamination level [10,11]. We showed that it was possible to identify *eae*-positive STEC from 2.59 × 10^8^ copies.mL^−1^ of STEC in the absence of other interfering *E. coli* strains. Nevertheless, this study was a proof-of-principle, and the presence of a diverse background flora, as well as the presence of multiple *E. coli* strains, may hamper the analysis [10]. Since the STEC contamination level can be low (below 100 cells), it is necessary to determine the STEC full characterization limit in the presence of background *E. coli* using an assembly-based metagenomics approach.

In this study, we artificially co-contaminated pasteurized milk using increasing and known inoculation levels of a commensal *E. coli* and an *eae*-positive STEC strain, both originally isolated from raw milk. Because the natural background flora of raw milk and the potential presence of commensal *E. coli* may affect the growth of STEC, pasteurized milk was used to better control the STEC:commensal ratios [10]. Each contaminated milk sample was enriched in acriflavine-supplemented buffered peptone water (BPW) before sequencing. We used the published STECmetadetector pipeline on MinION-generated data [10] and determined the limit of this approach.

## 2. Materials and Methods

### 2.1. E. coli Strains Used for Artificial Co-Contamination

Two *E. coli* strains were used in this study for artificial contamination of pasteurized milk. One commensal *E. coli* (*stx* and *eae* negative) strain was isolated from cow raw milk in 2021. This strain (BfR-EC-19174, *stx*- *eae*-, O2:H10, ST 6527) was isolated following the ISO/TS 13136:2012 method [4]. Its serogroup was determined using seroagglutination [12] and further confirmed with qPCR using primers and probes as described by Delannoy and colleagues [13]. The second strain, 6423-O26, was an *eae*-positive STEC of serotype O26:H11 as already described [10,14]. Both strains BfR-EC-19174 (referred to as the commensal *E. coli*) and 6423-O26 (referred to as the *eae*-positive STEC) were revived from 20–30% glycerol stock on TSA plates. One colony was cultivated in brain heart infusion (BHI) overnight at 37 °C with agitation.

### 2.2. Artificial E. coli Mixtures

For each strain, one milliliter of overnight pure culture was used as a starting material for eight serial dilutions (1:10) in BHI or BPW. Optical density (OD) was measured at 600 nm using 1 mL BHI or 1 mL of the following: 1 mL BHI in 9 mL BPW, as a blank. For inoculum determination, triplicate cell counting was performed by plating three dilutions on tryptic soy agar (TSA) plates (100 µL) and incubated overnight at 37 °C. Five mixtures of the two *E. coli* strains were prepared as represented in Table 1.

### 2.3. Co-Contamination of Pasteurized Milk

Pasteurized milk with 3.8% fat was bought from a grocery store in Berlin (Germany), and 1 mL was artificially contaminated with 1 mL of each mixture of commensal (BfR-EC-19174) and *eae*-positive STEC (6423-O26) prepared as mentioned in Table 1. For artificial contamination, three biological replicates of artificially contaminated milk samples were enriched for 18–20 h at 37 °C in 8 mL of BPW supplemented with acriflavine at a final concentration of 12 mg.L^−1^ [4].

### 2.4. DNA Extraction and Quality Control

One milliliter of milk-enriched mixtures was used for DNA extraction using the MasterPure Lucigen protocol as described by Jaudou et al., 2022 [10]. To ensure the feasibility of MinION sequencing, the quality of the extracted DNA was assessed using Nanodrop 1.0 (A260/A280 and A260/A230 ratios), and DNA was quantified using Qubit 3.0 and the broad range kit following the manufacturer’s instructions.

### 2.5. Detection and Quantification of Commensal and Eae-Positive STEC Strains Using qPCR and qdPCR, Respectively

Real-time PCR was performed to detect the presence of each strain used for the artificial contamination of pasteurized milk after enrichment and quantitative digital PCR (qdPCR) for quantitative estimation (number of copies.mL^−1^). The *wzx* gene coding for the O-antigen flippase is serogroup-specific and present in a single copy in the *E. coli* genome. Genetic markers *wzx*_O2_ and *wzx*_O26_ were used to detect and quantify the commensal and the *eae*-positive STEC, respectively, in each milk enrichment. Sequences for primers and probes are given in Table 2. Probes were labeled with 6-carboxyfluorescin (FAM) and black hole quencher (BHQ1) (Eurofins).

For real-time PCR, a mix (20 µL) containing 1X PerfeCTa qPCR ToughMix low ROX (QuantaBio), primers, and probes at a final concentration of 0.3 µM and completed with nuclease-free water was used for each marker. Extracted DNA (2 µL) was added to the mix. Targeted sequences were amplified using the CFX96 system (BioRad) with a first step at 95 °C for 10 min (5 °C.s^−1^), a second step consisting of 39 cycles at 95 °C for 15 s (2 °C.s^−1^) and at 60 °C for 1 min, and a final step at 40 °C for 30 s (5 °C.s^−1^).

Mixes for qdPCR were prepared as follows: 3 µL PerfeCTa 2X qPCR ToughMix low ROX (QuantaBio), 0.6 µL 20X GE sample loading reagent (Fluidigm), 0.3 µL 20X primer stock prepared using 18 µM for each primer (forward and reverse) and 4 µM of probe, and 1.8 µL of extracted DNA, completed to 6 µL with nuclease-free water. Quantitative digital PCR was run on qdPCR 37k IFC digital array microfluidic chips using a Biomark system (Standard BioTools). For amplification, the thermal profile constituted of a first step at 50 °C for 2 min and a second step at 95 °C for 10 min followed by 40 cycles at 95 °C for 15 sec and 60 sec at 60 °C (2 °C s^−1^). Data analysis was performed with the Fluidigm digital PCR analysis software v4.1.2.

Mixes as well as thermal profiles used for the two PCR methods are also detailed in [10]. Boiled DNA (from 1 mL of overnight culture, boiled at 95 °C for 10 min and centrifuged at 10 000 rpm for 5 min) from CB-16230 and BfR-EC-19174 strains was used as a positive control for *wzx*_O26_ and *wzx*_O2_, respectively. A non-template control was also included.

### 2.6. MinION Sequencing of Artificially Co-Contaminated Milk

Two biological replicates of each enrichment were sequenced using the MinION platform except for Mix3 for which all replicates were sequenced. Libraries were prepared using the SLK-SQK109 ligation sequencing and the EXP-NBD104 or EXP-NBD114 barcoding kits, as previously described [10]. Five to six samples were loaded on FLO-MIN106 R9.4.1 flow cells and sequenced using the Mk1C device without live basecalling or demultiplexing.

### 2.7. Sequencing Data Analysis

Raw data (fast5 files) were basecalled using guppy_basecaller v6.0.1 + 652ffd1 and the super high accuracy model including a q-score filter of 10. Data were demultiplexed using guppy_barcoder v6.0.1 + 652ffd1 and *–trim-adapters* and *–compress-fastq* parameters. Basecalled and demultiplexed data were processed using the STECmetadetector pipeline v0.1.2 [10] (https://gitlab.com/bfr_bioinformatics/STECmetadetector, accessed on 26 July 2023). Extracted *E. coli* reads in fastq format were deposited in NCBI BioProject PRJNA982778. We assumed that the inoculated STEC strain was fully characterized when the two virulence genes *stx* and *eae* were co-located on the same contig. The figures were generated using *ggplot2* v3.4.0, *reshape2* v1.4.4, and *patchwork* v1.1.2 packages on R v4.1.2 and R studio v2021.9.0.351 [16,17,18]. The O-antigen clusters for O2 and O50 differ by a maximum of 2 potential SNPs [13,19] and are therefore considered identical and treated as a single molecular serogroup [20]. However, *wzy*_O2_ and *wzy*_O50_ (1 SNP difference, which cannot be reliably differentiated using MinION sequencing) have separate entries in the database included in the STECmetadetector pipeline. On the contrary, there is a single entry for *wzx*_O2/O50_. In the mapping analyses, reads mapping on *wzy*_O2_ and *wzy*_O50_ were thus combined.

## 3. Results

### 3.1. Estimation of the Inoculation Level Using Cell Counting and Post-Enrichment Quantification Using qdPCR

In order to determine the STEC:commensal ratio that would prevent the full characterization of the *eae*-positive STEC strain, we used five mixes to contaminate pasteurized milk samples. In Mix 1, we used an equal ratio of STEC and commensal strain before enrichment. In Mixes 2 and 3, we used a commensal strain 10 times and 100 times in excess compared to the STEC strain, respectively. In Mixes 4 and 5, we used a STEC strain 10 times and 100 times in excess compared to the commensal strain, respectively. Actual STEC and commensal strain inoculation levels used for the five mixes were determined using plate counting on TSA. Results are reported in Table 3. Each mix was used to artificially contaminate pasteurized milk before enrichment at 37 °C in acriflavine-supplemented BPW. To estimate the growth of the two strains during enrichment, we used qdPCR on their respective O-group genetic marker (*wzx*_O26_ for the STEC and *wzx*_O2_ for the commensal). Quantification of the two strains after enrichment is also reported (Table 3).

The results show that the conditions used here for enrichment (37 °C with acriflavine) seemed to favor the growth of the STEC O26 strain compared to the O2 commensal strain (8.62 × 10^7^–5.94 × 10^8^ copies.mL^−1^, mean = 4.07 × 10^8^ copies.mL^−1^ for the STEC and 3.74 × 10^5^–2.29 × 10^8^ copies.mL^−1^, mean = 5.10 × 10^7^ copies.mL^−1^ for the commensal) regardless of the inoculation level of the commensal O2 strain (1.43 × 10^2^ to 1.39 × 10^4^ CFU.mL^−1^). The O26 STEC strain always reached the 10^8^ copies.mL^−1^ threshold previously determined as the minimal quantity required for a single strain assembly [10]. The STEC strain was quantified approximately 1 to 3 logs higher than the commensal strain in all mixtures except Mix 3 (inoculation STEC:commensal ratio of 1:100) (Table 3). In this condition (Mix 3), the commensal strain was inoculated at a concentration 100 times higher than the STEC, but quantification results on O2 and O26 strains were similar post-enrichment (1.66 × 10^8^ and 3.86 × 10^8^ copies.mL^−1^ for O2 and O26, respectively). Although the O2 inoculation level corresponded to an O26:O2 ratio of 1:221 (Table 3), the STEC strain still appeared to grow to a ratio that reached 2:1 (O26:O2) (Table 3).

### 3.2. Characterization of the STEC Strain in Presence of Commensal E. coli Using STECmetadetector

Two replicates of each condition and three replicates for the condition with equal levels of STEC:commensal post-enrichment (Mix 3) were sequenced using MinION sequencing, and data were analyzed using the STECmetadetector pipeline.

**Figure 1:**
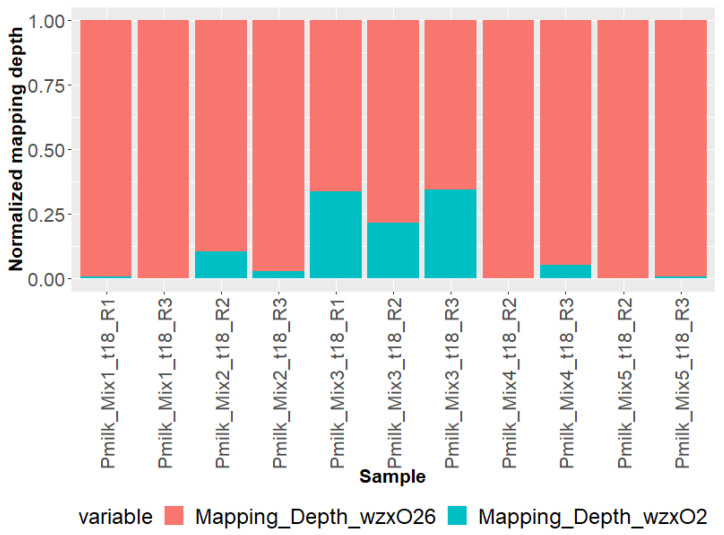
*E. coli* reads mapping depth on *wzx/wzy_O26_* and *wzx/wzy_O2_* genetic markers determined using STECmetadetector pipeline.

The STECmetadetector pipeline includes an initial mapping step to detect the presence of multiple O-groups in the set of *E. coli* reads. Figure 1 represents the read mapping depth on O-groups (*wzx* and *wzy* genetic markers) for O26 and O2. Mapping results were in line with quantification results (qdPCR) showing a higher number of reads mapped to O26 compared to O2. When the O2 strain was quantified below 10^8^ copies.mL^−1^ in the enriched samples (Mixes 1, 2, 4, and 5), only a few reads corresponding to O2 could be detected. The low read count attributable to the O2 strain explains the apparent ratio difference between qdPCR and mapping, especially in Mixes 4 and 5.

**Figure 2:**
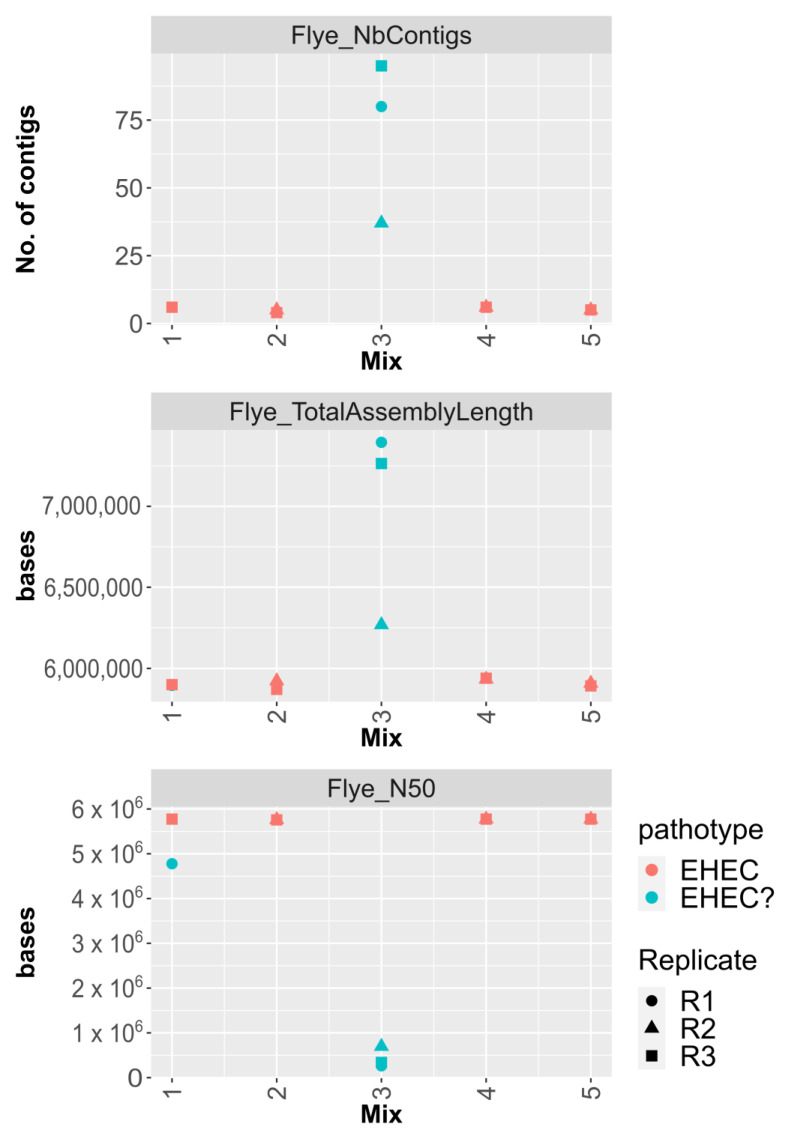
Metrics of assemblies generated using Flye on extracted *E. coli* reads obtained from co-contamination of a commensal *E.coli* and STEC in pasteurized milk. The number of contigs, total assembly length (in bases), and the assembly N50 value are represented for each Mix. Mix 1 corresponds to co-contamination using equal proportions of the two strains and Mixes 2 and 3 to inoculation levels with 10 times and 100 times higher concentration of commensal, while Mixes 4 and 5 corresponds to inoculation levels with 10 times and 100 times higher concentration of STEC, respectively. The biological replicate (R1, R2, and R3) is represented by a specific symbol. A red color represents successful characterization of the STEC strain while a turquoise color represents when *stx* and *eae* were not identified on the same contig.

Assemblies of *E. coli* reads were performed using Flye included in the STECmetadetector pipeline. The inoculated STEC strain was considered fully characterized when the two virulence genes *stx* and *eae* were co-localized on the same contig. Figure 2 shows that the *eae*-positive O26 STEC was characterized in 7/11 samples of artificially co-contaminated pasteurized milk. For all of those samples, the chromosome was in one contig with *stx*, *eae,* and serotype genetic markers (*wzx/wzy*_O26_ and *fliC*_H11_) co-localized (Figure 2). The STEC strain was successfully characterized using the STECmetadetector pipeline in all samples in which the O26 strain attained 10^8^ copies.mL^−1^ and the O26:O2 ratio (determined by qdPCR or mapping) was at least 10:1. The only exception was for one replicate of Mix 1 (Pmilk_Mix1_R1) in which O26 was quantified to 8.62 × 10^7^ copies.mL^−1^ after enrichment (Supplementary File S1).

When the STEC and commensal strains were in equivalent quantities post-enrichment (i.e., Mix3), the O26 *eae*-positive STEC strain could not be characterized, even though it reached 10^8^ copies.mL^−1^. All assemblies from these samples (Pmilk_Mix3) were highly fragmented (Figure 2). In this condition, the STEC:commensal ratio after enrichment was 2:1 as quantified using qdPCR and below 10:1 as determined using mapping (Table 3 and Supplementary File S2).

## 4. Discussion

LEE-positive STEC constitute the STEC subgroup most frequently associated with severe human symptoms such as hemorrhagic colitis or HUS. Therefore, methods for detecting STEC in food samples often include the detection of both *stx* and *eae* genes by qPCR followed by the characterization of an isolated strain [4]. The characterization of STEC strains detected in contaminated food products is important in order to evaluate their pathogenic potential, but the isolation of a STEC strain from food products is often unsuccessful.

In previous work, we developed long-read metagenomics as an approach to characterize STEC strains from raw milk in an isolation-free manner. We have shown that STEC characterization directly from raw milk is possible but is hindered by the presence of background flora and multiple *E. coli* strains [10]. Cattle are asymptomatic carriers of STEC and can carry from 10 to 10^9^ CFU of STEC per gram of feces [21,22]. The main source of milk contamination comes from cattle feces in which not only STEC but also different *E. coli* strains may be present simultaneously [23]. To the best of our knowledge, there is no study that assesses the ratio of STEC to other *E. coli* in milk samples or cattle feces samples. Although we have shown that long-read metagenomics is suitable for characterizing STEC in complex matrices such as raw milk, it is important to assess the limit of background *E. coli* that would impede the characterization of the STEC using the STECmetadetector pipeline.

Here, we aimed at determining the ratio of commensal *E. coli* to STEC that would prevent the characterization of STEC. Background flora may limit the growth of the *E. coli* strains, which leads to lower output of *E. coli* reads. In our previous work, we used acriflavine, an antibiotic targeting Gram-positive bacteria, to handle the influence of background flora limiting *E. coli* strains growth [4,10]. In this study, even though we do not have background flora in pasteurized milk, we used the same enrichment conditions, including the use of acriflavine as it may potentially affect the growth of STEC and non-pathogenic *E. coli* differently [10,24].

We had previously determined that *eae*-positive STEC could be characterized using long-read metagenomics and an assembly-based approach from 2.59 × 10^8^ copies.mL^−1^ post-enrichment in the absence of commensal *E. coli* flora in the milk [10]. The results obtained in this study were in line with what was determined before and allowed us to refine the characterization threshold. Overall, we estimated that a minimum of 10^8^ copies.mL^−1^ of STEC is required to achieve STEC characterization. Indeed, here, samples where the *eae*-positive STEC strain was in a 10 times excess over the commensal strain and quantified above 10^8^ copies.mL^−1^ post-enrichment were characterized. In addition, samples in which the commensal strain was below 10^8^ copies.mL^−1^ post-enrichment yielded very few reads corresponding to this strain, which then failed to assemble. The commensal strain reached the characterization threshold only when inoculated 100 times in excess of the STEC strain but resulted in a ratio of 2:1 STEC:commensal post-enrichment. As a consequence, it appears that the STEC:commensal ratio is another important factor to evaluate.

In all pasteurized milk samples artificially co-contaminated, the inoculated *eae*-positive STEC strain was characterized when it was quantified above 10^8^ copies.mL^−1^ post-enrichment and at least one log higher than the commensal strain. The exception was for one replicate inoculated with Mix 1 in which the estimated level of STEC determined using qdPCR was slightly below the 10^8^ copies.mL^−1^ threshold. The limiting condition for characterizing the STEC strain appears to be when the two strains grew to an equivalent ratio post-enrichment.

It is noteworthy that the enrichment conditions seem to affect the growth of the two *E. coli* strains differently. Indeed, the ratio of STEC:commensal has evolved drastically after enrichment from the inoculated levels. The STEC strain was quantified to higher levels than the commensal strain post-enrichment, even when inoculated 10 times less. Owing to their cell wall structure and the presence of the AcrB efflux pump, *E. coli* should not be sensitive to acriflavine [25]. In our previous work, we compared the growth of the STEC strain used in this study (6423-O26) in the presence and absence of acriflavine and observed no negative impact of acriflavine on the growth of this strain. It is, however, possible that the acriflavine used during enrichment affected the growth of the commensal *E. coli* strain (BfR-EC-19174). Some studies have already observed a strain-dependent effect of enrichment conditions [24,26,27]. Another option is a possible competition between the STEC and the commensal *E. coli* strain, with an advantage for the STEC growth. However, analysis of the genome sequences of both strains indicated the presence of multiple bacteriocins, although the level of expression of these bacteriocins was not examined [28].

Despite the possible influence of the enrichment conditions on the growth of the two strains, the limits that were determined in this study are more related to the post-enrichment steps, including DNA extraction, library preparation, data acquisition, and data processing [29]. It has been shown that the DNA extraction procedure can affect the structure of the population, especially in these particular conditions where high quantities of high-molecular-weight DNA are required (MinION sequencing from complex matrices) [30]. Similarly, according to the properties of the DNA (for example, GC content), the library preparation step performs differently [31,32,33]. Both DNA extraction and the performance of library preparation steps constitute the first bias that may lead to a different population structure compared to the original sample [29,34,35]. The second main bias is introduced by the sequencing technology and the data processing part. MinION sequencing does not provide a homogenous quantity of data, read length, or quality across flow cells and samples. Some studies have demonstrated that high-GC species are under-represented in sequencing reads and contain a higher error rate [36,37]. In addition, the removal of the shortest reads and low-quality data during filtering steps might increase the bias in the population structure.

In this study, we used the depth of read mapping to the O-group genetic markers to estimate the O26:O2 ratio and check whether it differed from the ratio observed using qdPCR post-enrichment. The ratio determined using qdPCR and mapping was in a similar order of magnitude for samples of Mixes 1, 2, and 3, whereas the ratio observed for Mix 4 and Mix 5 were 100 and 10 times higher, respectively, using qdPCR (O26:O2 of 1000:1) than using the mapping approach (ratios of 10:1 and 100:1, respectively, for each mix). Considering the difference in proportion between the two strains, as estimated using qdPCR, the required amount of sequencing data could not be generated to reach a similar ratio of 1000:1. Our results suggest that the different post-enrichment steps performed here did not influence the O26:O2 ratio.

The main limitation of our approach concerns the assembly process. Indeed, when the two strains reached the required level for full characterization but were present at an equivalent ratio (Mix3 O26:O2 ratio below 10:1), the assembler failed to distinguish the STEC from the commensal strain. Current long-read assemblers (even those allowing metagenomics assemblies) are not able to differentiate different strains from the same species (less than 5% ANI divergence) since they share many genomic regions [38,39]. Although different strains will behave differently during the enrichment step, we showed that to be characterized, the amount of STEC data should be at least 10 times in excess compared to the commensal strain. It is important to note that six samples were multiplexed per MinION flow cell. Different multiplexing conditions would lead to different thresholds.

## 5. Conclusions

Overall, this study refined the limit of the long-read metagenomics approach developed in previous work to characterize *eae*-positive STEC in raw milk. In the conditions of this study, we observed that an *eae*-positive STEC strain can be characterized without the need to isolate the strain if the STEC strain can grow at least 10 times in excess compared to background *E. coli* strains post-enrichment, provided that the background flora does not prevent it from reaching the 10^8^ copies.mL^−1^. Additionally, this study highlights the need for strain-aware assemblers or alternative approaches that would help identify different strains from a single species.

Under certain conditions, isolation-independent approaches are promising and could be applied in the case of ambiguous results that render decision making difficult, particularly when the STEC strain cannot be isolated. Many studies on STEC prevalence in cattle have been conducted but none on STEC prevalence compared to background *E. coli* [40,41]. Such studies would give insights into the application of long-read metagenomic approaches to characterize STEC among other *E. coli.*

## Figures and Tables

**Table 1:** Commensal *E. coli* and *eae*-positive STEC artificial mixtures used for co-contamination of pasteurized milk.

Mix	O26:O2 Estimated Ratio	STEC Inoculum (X CFU.mL^−1^)	Commensal Inoculum (X CFU.mL^−1^)	Final STEC:Commensal Estimated Inoculum in Milk (X CFU.mL^−1^)
1	1:1	10^2^	10^2^	5:5
2	1:10	10^2^	10^3^	5:50
3	1:100	10^2^	10^4^	5:500
4	10:1	10^3^	10^2^	50:5
5	100:1	10^4^	10^2^	500:5

**Table 2:** Primers and probes sequences for *wzx*_O2_ and *wzx*_O26._

Genetic Marker	Sequence (5′-3′)	Reference
*wzx*_O2_	Primer F- GCCAAGTGCAAAGTTTAATCACAAT Primer R- CTTGCCAATTTTCCGCAGTATAT Probe [6FAM]- CCTCTGCACCTGTAAGCACTGGCCTT-[BHQ1]	[13]
*wzx*_O26_	Primer F- CGCGACGGCAGAGAAAATT Primer R- AGCAGGCTTTTATATTCTCCAACTTT Probe [6FAM]-CCCCGTTAAATCAATACTATTTCACGAGGTTGA-[BHQ1]	[15]

**Table 3:** Estimated inoculum (CFU.mL^−1^) and relative quantification (qdPCR, copies.mL^−1^) of the commensal and *eae*-positive STEC strain after enrichment of artificially co-contaminated milk.

Sample	Desired Inoculum O26:O2 Ratio	O2 Inoculum Estimated Using Cell Counting (CFU.mL^−1^)	O26 Inoculum Estimated Using Cell Counting (CFU.mL^−1^)	Estimated O26:O2 Ratio	Relative Quantification of O2 after Enrichment Using qdPCR (*wzx*_O2_, copies.mL^−1^)	Relative Quantification of O26 after Enrichment Using qdPCR (*wzx*_O26_, copies.mL^−1^)	O26:O2 Ratio Post-Enrichment
Pmilk_Mix1	1:1	1.43 × 10^2^	6.30 × 10^1^	1:2	1.48 × 10^6^	2.59 × 10^8^	175:1
Pmilk_Mix2	1:10	1.39 × 10^3^	6.30 × 10^1^	1:22	3.55 × 10^7^	4.81 × 10^8^	13:1
Pmilk_Mix3	1:100	*1.39 × 10^4^*	6.30 × 10^1^	1:221	1.66 × 10^8^	3.86 × 10^8^	2:1
Pmilk_Mix4	10:1	1.43 × 10^2^	6.00 × 10^2^	4:1	4.08 × 10^5^	4.57 × 10^8^	1119:1
Pmilk_Mix5	100:1	1.43 × 10^2^	*6.00 × 10^3^*	41:1	2.53 × 10^5^	3.46 × 10^8^	1367:1

Data in italics indicate that the number of cells was extrapolated using cell counting results of previous dilutions.

## Data Availability

The STECmetadetector pipeline is freely available at https://gitlab.com/bfr_bioinformatics/STECmetadetector, accessed on 26 July 2023). The extracted *Escherichia coli* reads from each sample were deposited on NCBI under BioProject PRJNA982778 available at http://www.ncbi.nlm.nih.gov/bioproject/982778, accessed on 26 July 2023.

## Supplementary Materials

The following supporting information can be downloaded at: https://www.mdpi.com/article/10.3390/microorganisms11082043/s1. File S1: Relative quantification (qdPCR, copies.mL^−1^) of the commensal (O2) and *eae*-positive STEC (O26) strain after enrichment of artificially co-contaminated milk. File S2: Reads mapping depth on the commensal (O2) and on the *eae*-positive STEC (O26) genetic marker from the *E. coli* set of reads.
